# Assessing the Applicability of Cardiac Myosin Inhibitors for Hypertrophic Cardiomyopathy Management in a Large Single Center Cohort

**DOI:** 10.31083/j.rcm2506225

**Published:** 2024-06-20

**Authors:** Ali Amr, Elham Kayvanpour, Christoph Reich, Jan Koelemen, Shamily Asokan, Norbert Frey, Benjamin Meder, Farbod Sedaghat-Hamedani

**Affiliations:** ^1^Institute for Cardiomyopathies & Center for Cardiogenetics, Department of Medicine III, University of Heidelberg, 69120 Heidelberg, Germany; ^2^DZHK (German Centre for Cardiovascular Research), 69120 Heidelberg, Germany; ^3^Stanford Genome Technology Center, Stanford University School of Medicine, Palo Alto, CA 94305, USA

**Keywords:** hypertrophic cardiomyopathy, cardiac myosin inhibitors, Aficamten, Mavacamten

## Abstract

**Background:**

Cardiac myosin inhibitors (CMIs), including Mavacamten and Aficamten, have emerged as a groundbreaking treatment for hypertrophic cardiomyopathy (HCM). The results from phase 2 and 3 randomized clinical trials for both drugs have showed promising outcomes. However, the highly selective patient recruitment for these trials raises questions about the generalizability of the observed positive effects across broader patient populations suffering from HCM.

**Methods:**

A retrospective cohort study at University Hospital Heidelberg included 404 HCM patients. Baseline assessments included family history, electrocardiograms (ECGs), and advanced cardiac imaging, to ensure the exclusion of secondary causes of left ventricular hypertrophy.

**Results:**

Among the HCM patients evaluated, only a small percentage met the inclusion criteria for recent CMI trials: 10.4% for EXPLORER-HCM and 4.7% for SEQUOIA-HCM. The predominant exclusion factor was the stringent left ventricular outflow tract (LVOT) gradient requirement.

**Conclusions:**

This study highlights a significant discrepancy between patient demographics in clinical trials and those encountered in routine HCM clinical practice. Despite promising results from the initial randomized clinical trials that led to the approval of Mavacamten, the selected patient population only represents a small part of the HCM patient cohort seen in routine clinics. This study advocates for further expanded randomized clinical trials with broader inclusion criteria to represent diverse primary HCM patient populations.

## 1. Introduction

Primary hypertrophic cardiomyopathy (HCM) is a genetic myocardial disorder [1]. It is characterized primarily by significant left ventricular hypertrophy that is not attributable to other cardiovascular conditions, leading to disrupted cardiac function and a wide range of possible symptoms [2]. The pathophysiology of HCM navigates through a spectrum that encompasses an asymmetrical septal hypertrophy, potentially leading to left ventricular outflow tract obstruction, to a more diffused variant affecting the entirety of the left ventricle. This heterogeneity in clinical presentations underscores not only the variable symptomatology—ranging from benign to severe heart failure, atrial fibrillation, and sudden cardiac death—but also instigates challenges in management and therapeutic strategies [3, 4].

Hypertrophic obstructive cardiomyopathy (HOCM) treatment guidelines have until recently been based on general medications that modulate heart rate and left ventricular contractility, and on invasive methods like septal reduction therapy [1]. However, these treatments often fall short in efficacy and could possibly cause side effects or complications [5]. HCM is frequently a progressive disease, and even with optimal care, poses significant long-term health problems [6]. For these reasons, therapeutic strategies that specifically address the fundamental disease mechanisms of HCM represent a significant requirement. The development of cardiac myosin inhibitors (CMIs) is the first step in this direction. CMIs are a novel class of drugs that act as small-molecule allosteric inhibitors of cardiac myosin, affecting myocardial contractility [6]. There are currently two CMIs that are available for clinical use after the completion of Phase 3 clinical trials, Mavacamten from Bristol Myers Squibb and Aficamten from Cytokinetics [7, 8, 9, 10]. Several randomized clinical studies have demonstrated the positive effects of CMIs on quality of life, exercise capacity, left ventricular outflow tract (LVOT) gradient, cardiac biomarkers and diastolic function [8, 11]. Based on the results of these clinical trials, Mavacamtan has now received regulatory approval in Europe and the United States [12].

The recent randomized clinical trials (RCTs) evaluating CMIs have yielded promising clinical outcomes, yet the patient selection process has been notably stringent and selective [10, 13, 14]. RCTs for Mavacamten and Aficamten implemented exclusion criteria that significantly narrowed the pool of HCM patients suitable for these novel therapies. Table 1 (Ref. [13, 14]) summarizes the inclusion and exclusion criteria for both clinical investigations. The representativeness of the trial cohorts in comparison to the broader HCM patient demographic typically encountered in clinical practice could be put into question. This study aims to highlight the need for further clinical investigations encompassing a broader range of HCM patients, thereby improving the generalizability and applicability of CMI treatments.

**Table 1.** **Inclusion and exclusion criteria of EXPLORER-HCM and SEQUOIA-HCM clinical trials [13, 14]**.

EXPLORER-HCM clinical trial investigating Mavacamten	
Key inclusion criteria	Key exclusion criteria
Aged at least 18 years	History of syncope or sustained ventricular tachyarrhythmia with exercise within the past 6 months
Diagnosed with obstructive HCM (LV hypertrophy with max LV wall thickness ≥15 mm or ≥13 mm if familial HCM)	QT interval corrected >500 ms using Fridericia’s formula
Peak LVOT gradient at least 50 mmHg (at rest, after Valsalva manoeuvre or exercise)	Paroxysmal or intermittent atrial fibrillation present on screening ECG
Left ventricular ejection fraction (LVEF) at least 55%	Persistent or permanent atrial fibrillation not on anticoagulation for 4 weeks+ or not adequately rate-controlled within the past 6 months
NYHA class II–III symptoms and able to perform upright CPET	Patients on stable doses of β blockers or calcium channel blockers for at least 2 weeks before screening, without anticipated changes during the study (except disopyramide)
SEQUOIA-HCM clinical trial investigating Aficamten	
Key inclusion criteria	Key exclusion criteria
Adults between 18 and 85 years of age at screening	Aortic stenosis or fixed subaortic obstruction or moderate-severe mitral regurgitation not due to systolic anterior motion of the mitral valve
Body weight ≥35 kg at screening	Known infiltrative or storage disorder causing cardiac hypertrophy that mimics HOCM (e.g., Noonan syndrome, Fabry disease, amyloidosis)
Diagnosed with HOCM per the following criteria:	History of LV systolic dysfunction (LVEF <45%) at any time during their clinical course
(a) LV hypertrophy and non-dilated LV chamber in the absence of other cardiac disease	
(b) Minimal wall thickness ≥15 mm (minimal wall thickness ≥13 mm is acceptable with positive family history of HCM or with known disease-causing gene mutation)	
Adequate acoustic windows for echocardiography	Inability to exercise on a treadmill or bicycle
Resting LVOT gradient ≥30 mmHg and <50 mmHg AND post-Valsalva LVOT gradient ≥50 mmHg	Has been treated with septal reduction therapy (surgical myectomy or percutaneous alcohol septal ablation) or plans for either treatment during the study period
LVEF ≥60% at screening	History of syncope or sustained ventricular tachyarrhythmia with exercise within 6 months before screening
NYHA functional class II or III at screening	Has received prior treatment with Aficamten or Mavacamten
Patients on beta-blockers, verapamil, diltiazem, or ranolazine should have been on stable doses for >6 weeks prior to randomization and anticipate remaining on the same medication regimen during the study	Paroxysmal atrial fibrillation or flutter documented during the screening period
Hemoglobin ≥10 g/dL at screening	Paroxysmal or permanent atrial fibrillation is only excluded if: (1) rhythm restoring treatment has been required ≤6 months prior to screening or (2) rate control and anticoagulation have not been achieved for at least 6 months prior to screening

CPET, cardiopulmonary exercise stress testing; ECG, electrocardiogram; HOCM, hypertrophic obstructive cardiomyopathy; NYHA, New York Heart Association; LVEF, left ventricular ejection fraction; LVOT, left ventricular outflow tract; HCM, hypertrophic cardiomyopathy; LV, left ventricular.

## 2. Methods

The study was retrospective and single-center to evaluate individuals diagnosed with HCM. Compliance with institutional protocols was maintained during clinical assessments, diagnostics, and monitoring, respecting the guidelines set by the Declaration of Helsinki. Ethical approval was obtained from the University of Heidelberg Medical Faculty’s ethics committee. Consecutive patients presenting with primary HCM at the University Hospital Heidelberg from 2001 to 2017 were included in the study. Diagnostic criteria for HCM were a left ventricular wall thickness of ≥15 mm without a discernible cause, or meeting the established diagnosis criteria for familial HCM. Exclusion criteria were secondary hypertrophy due to conditions such as persistent uncontrolled hypertension, valvular heart disease, inflammatory or systemic diseases, and any syndromic or metabolic disorders.

Enrolled patients were subject to an extensive clinical workup, which entailed gathering detailed familial medical histories, performing electrocardiograms (ECGs), echocardiograms, blood tests, and exercise stress tests. Additional cardiac examinations including cardiac magnetic resonance imaging (MRI), left ventricular biopsy, and coronary angiography were utilized as necessary to rule out secondary etiologies. The patients underwent comprehensive phenotyping to differentiate between obstructive, non-obstructive and secondary HCM. The results from all the clinical examinations were examined by specialists in our cardiomyopathy outpatient clinic. In cases where a latent obstruction in LVOT was suspected, the patients either underwent exercise echocardiography or invasive pressure measurements under Valsalva or provocation during their heart catheterization, as per the standard operating procedure (SOP) set by our center. Furthermore, all patients received a Holter-monitor ECG for 24 hours at least once every two years to screen for arrhythmia, especially atrial fibrillation and ventricular tachycardias (VTs).

In this study, statistical analyses were conducted using R (version 4.1.2, GNU General Public License, https://www.r-project.org/), focusing on descriptive methods to characterize the HCM patient population. The descriptive statistics provided a comprehensive overview of the baseline demographics, clinical presentations, and treatment modalities among the cohort. These analyses encompassed calculations of mean and standard deviation for continuous and categorical variables. Additionally, the study employed Fisher’s exact test to assess the differences in β-blocker therapy administration between obstructive and non-obstructive HCM patients, using a *p*-value of under 0.05 to indicate statistical significance.

## 3. Results

This study comprised a final cohort of 404 HCM patients from the cardiomyopathy outpatient clinic at the University Hospital Heidelberg. Table 2 details the baseline demographics of the patient population. The cohort predominantly consisted of males (67%), and the average age was 47.33 ± 18.28 years. A significant proportion (38%) reported a family history of cardiomyopathy among first-degree relatives. Most of the patients presented with minimal heart failure symptoms, as indicated by 93% of the patients being classified as New York Heart Association (NYHA) class I and II. Obstructive HCM was documented in 31% of patients at the initial presentation. The mean LVOT gradient in those with obstructive HCM was 58.67 ± 47.35 mmHg. There were no marked differences in the administration of β-blocker therapy between HOCM and non-obstructive HCM (HNCM) patients (67% vs 69%; *p* = 0.71).

**Table 2.** **Patient demographics**.

Patient characteristics at baseline		Values
Patients, number (total)		404
Female, number (%)		133 (33)
Phenotype		
	HNCM, number (%)	278 (69)
	HOCM, number (%)	126 (31)
Age at diagnosis, mean ± SD, years		47.33 ± 18.28
Heart rate, mean ± SD, beats/min		70.62 ± 15.53
Blood pressure		
	Systolic, mean ± SD, mmHg	126.3 ± 21.11
	Diastolic, mean ± SD, mmHg	77.65 ± 13.12
Left bundle-branch block, number (%)		45 (11)
NYHA I, number (%)		180 (45)
NYHA II, number (%)		196 (49)
NYHA III, number (%)		28 (7)
NYHA IV, number (%)		0 (0)
6MWT, mean ± SD, m		508.15 ± 110.77
Atrial fibrillation, number (%)		103 (25)
Laboratory results		
	NT-proBNP, median (1Q; 3Q), ng/L	609 (225; 1280)
	hs-TNT, median (1Q; 3Q), pg/L	13 (6; 24)
Echocardiography		
	LV ejection fraction, mean ± SD, %	53.65 ± 7.16
	Max. LA diameter, median (1Q; 3Q), mm	44 (36; 48)
	Max. LV wall thickness, median (1Q; 3Q), mm	18 (15.8; 23.2)
	Max. LVOT gradient, mean ± SD, mmHg	27.8 ± 42.2

Baseline characteristics of the patient population. SD, standard deviation; HNCM, hypertrophic non-obstructive cardiomyopathy; HOCM, hypertrophic obstructive cardiomyopathy; NYHA, New York Heart Association; 6MWT, 6-minute-waling-test; NT-proBNP, N-terminal prohormone of brain natriuretic peptide; hs-TNT, high-sensitive troponin T; LV, left ventricular; LA, left atrium; LVOT, left ventricular outflow tract.

**Fig. 1.**
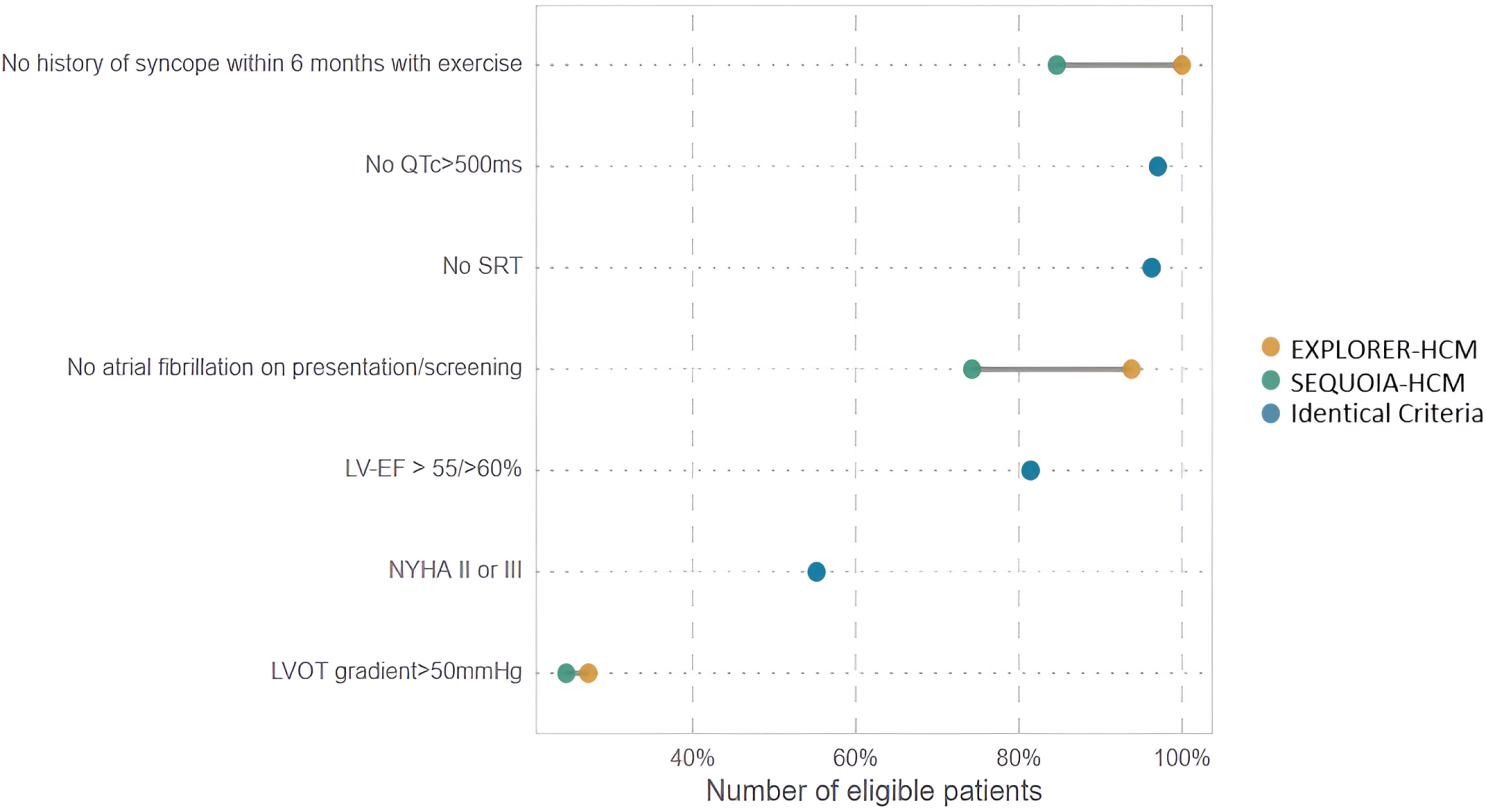
**Proportion of HCM patient cohort meeting clinical trial eligibility criteria.** This figure displays the eligibility of 404 hypertrophic cardiomyopathy (HCM) patients for the EXPLORER-HCM and SEQUOIA-HCM clinical trials. The x-axis represents the percentage of the cohort fulfilling specific criteria, while the y-axis lists these criteria, such as absence of syncope, QTc interval, presence of atrial fibrillation, left ventricular ejection fraction (LVEF), New York Heart Association (NYHA) class, and left ventricular outflow tract (LVOT) gradient. Each criterion is color-coded to indicate the proportion of patients eligible for the EXPLORER-HCM trial (orange), SEQUOIA-HCM trial (green), or those meeting identical criteria for both (blue). This graphical representation underscores the stringent selection process for these trials, particularly with respect to the LVOT gradient threshold. SRT, septal reduction therapy.

The cohort was further assessed for eligibility based on the inclusion and exclusion criteria of the Mavacamten and Aficamten clinical trials. Only 10.4% met the criteria for the EXPLORER-HCM study, and 4.7% for the SEQUOIA-HCM study. Even when only considering the patients with the obstructive form of HCM, just 25% and 7.1% met the criteria for EXPLORER-HCM and SEQUOIA-HCM respectively. The primary factor for exclusion among symptomatic patients (NYHA class II or higher) was the stringent LVOT gradient threshold, with 73% failing to meet the LVOT gradient requirement for EXPLORER-HCM and 75% for SEQUOIA-HCM. Furthermore, the clinical study on Aficamten required a resting gradient exceeding 30 mmHg and a post-Valsalva gradient above 50 mmHg (Fig. 1).

## 4. Discussion

Advancements in disease knowledge and pathophysiology have identified that sarcomere proteins are the fundamental cause of the hypertrophied myocardium in HCM [15]. This insight into the molecular underpinnings of HCM has given rise to the development of cardiac myosin inhibitors [5]. Among these, Mavacamten has achieved Food and Drug Administration (FDA) approval for the management of obstructive HCM [12]. The promising outcomes associated with CMIs are noteworthy, especially considering the historical reliance on beta blockers, calcium channel blockers, and disopyramide in the absence of evidence from randomized trials [6]. The current rigorous investigation of this novel class of therapy as a treatment for HCM patients represents a significant advancement. However, it is important to acknowledge that current studies are manufacturer funded and the patient selection does not necessarily reflect the wider spectrum of HCM patients commonly seen in clinical practice.

The results of this study underscore this point. In a HCM patient population (n = 404) that has been consecutively enrolled over a 10 year period, only 10.3% of the patients met the criteria of the Explorer-HCM study, on which the FDA approval was based. The European Commission has also approved Mavacamten for all obstructive HCM patients who are symptomatic (NYHA II/III) without specifically excluding patients that have not been represented in EXPLORER-HCM. REDWOOD-HCM and SEQUOIA-HCM were planned with even more stringent criteria regarding LVOT gradient so that less than 5% of the HCM patient population would have been eligible for the study. Furthermore, the REDWOOD or SEQUOIA criteria excluded all patients who had undergone any form of septal reduction therapy (SRT). This excluded a large cohort of patients, considering that most patients who are symptomatic under optimal medical therapy and present a LVOT gradient greater than 50 mmHg are likely to be offered a form of SRT to relieve symptoms.

Both Mavacamten and Aficamten drugs are currently being investigated in further clinical trials (MAPLE-HCM and ODYSEE-HCM) [16]. Nonetheless, our findings highlight the need for further randomized clinical studies to explore the benefits of this novel therapy in a broader and more representative HCM patient population.

## 5. Conclusions

In conclusion, the emergence of CMIs, specifically Mavacamten and Aficamten, represents a significant leap forward in the treatment of HCM, offering a new horizon of therapeutic possibilities. Despite the promising outcomes from initial clinical trials, our study reveals a stark discrepancy in patient selection criteria, highlighting that only a small fraction of the broader HCM population might benefit under the current guidelines. This underlines the urgent need for additional, more inclusive studies to truly gauge the effectiveness and applicability of these novel treatments across the full spectrum of HCM patients. Ensuring that future research encompasses a wider range of patient demographics will be crucial in making these groundbreaking therapies accessible and beneficial to a larger segment of the HCM community, thus optimizing patient care and outcomes in this complex and diverse patient population.

## Data Availability

Due to privacy and ethical considerations surrounding sensitive, personally identifiable information contained within our research data, we cannot offer open access to these materials. However, specific data inquiries can be addressed to the corresponding author, subject to strict compliance with participant confidentiality and privacy protection.
