# Correction: Amr et al. Assessing the Applicability of Cardiac Myosin Inhibitors for Hypertrophic Cardiomyopathy Management in a Large Single Center Cohort. Reviews in Cardiovascular Medicine. 2024; 25(6): 225

**DOI:** 10.31083/RCM56238

**Published:** 2026-07-23

**Authors:** Ali Amr, Elham Kayvanpour, Christoph Reich, Jan Koelemen, Shamily Asokan, Norbert Frey, Benjamin Meder, Farbod Sedaghat-Hamedani

**Affiliations:** ^1^Institute for Cardiomyopathies & Center for Cardiogenetics, Department of Medicine III, University of Heidelberg, 69120 Heidelberg, Germany; ^2^DZHK (German Centre for Cardiovascular Research), 69120 Heidelberg, Germany; ^3^Stanford Genome Technology Center, Stanford University School of Medicine, Palo Alto, CA 94305, USA

The authors would like to correct Table 1 (Ref. [1,2]) of Assessing the Applicability of Cardiac Myosin Inhibitors for Hypertrophic Cardiomyopathy Management in a Large Single Center Cohort [3], as errors were introduced in Table 1. The authors declare that these corrections do not change the result or conclusion of this paper. We sincerely apologize for having this error in the article, and apologize for any inconvenience caused. The authors have provided a corrected version of Table 1 (Ref. [4,5])here:

**The original Table 1:**

**Table 1.** **Inclusion and exclusion criteria of EXPLORER-HCM and SEQUOIA-HCM clinical trials [1,2]**.

EXPLORER-HCM clinical trial investigating Mavacamten	
Key inclusion criteria	Key exclusion criteria
Aged at least 18 years	History of syncope or sustained ventricular tachyarrhythmia with exercise within the past 6 months
Diagnosed with obstructive HCM (LV hypertrophy with max LV wall thickness ≥15 mm or ≥13 mm if familial HCM)	QT interval corrected >500 ms using Fridericia’s formula
Peak LVOT gradient at least 50 mmHg (at rest, after Valsalva manoeuvre or exercise)	Paroxysmal or intermittent atrial fibrillation present on screening ECG
Left ventricular ejection fraction (LVEF) at least 55%	Persistent or permanent atrial fibrillation not on anticoagulation for 4 weeks+ or not adequately rate-controlled within the past 6 months
NYHA class II–III symptoms and able to perform upright CPET	Patients on stable doses of β blockers or calcium channel blockers for at least 2 weeks before screening, without anticipated changes during the study (except disopyramide)
SEQUOIA-HCM clinical trial investigating Aficamten	
Key inclusion criteria	Key exclusion criteria
Adults between 18 and 85 years of age at screening	Aortic stenosis or fixed subaortic obstruction or moderate-severe mitral regurgitation not due to systolic anterior motion of the mitral valve
Body weight ≥35 kg at screening	Known infiltrative or storage disorder causing cardiac hypertrophy that mimics HOCM (e.g., Noonan syndrome, Fabry disease, amyloidosis)
Diagnosed with HOCM per the following criteria: (a) LV hypertrophy and non-dilated LV chamber in the absence of other cardiac disease. (b) Minimal wall thickness ≥15 mm (minimal wall thickness ≥13 mm is acceptable with positive family history of HCM or with known disease-causing gene mutation)	History of LV systolic dysfunction (LVEF <45%) at any time during their clinical course
Adequate acoustic windows for echocardiography	Inability to exercise on a treadmill or bicycle
Resting LVOT gradient ≥30 mmHg and <50 mmHg AND post-Valsalva LVOT-G ≥50 mmHg	Has been treated with septal reduction therapy (surgical myectomy or percutaneous alcohol septal ablation) or plans for either treatment during the study period
LVEF ≥60% at screening	History of syncope or sustained ventricular tachyarrhythmia with exercise within 6 months before screening
NYHA functional class II or III at screening	Has received prior treatment with Aficamten or Mavacamten
Patients on beta-blockers, verapamil, diltiazem, or ranolazine should have been on stable doses for >6 weeks prior to randomization and anticipate remaining on the same medication regimen during the study	Paroxysmal atrial fibrillation or flutter documented during the screening period
Hemoglobin ≥10 g/dL at screening	Paroxysmal or permanent atrial fibrillation is only excluded if: (1) rhythm restoring treatment has been required ≤6 months prior to screening or (2) rate control and anticoagulation have not been achieved for at least 6 months prior to screening

CPET, cardiopulmonary exercise stress testing; ECG, electrocardiogram; HOCM, hypertrophic obstructive cardiomyopathy; NYHA, New York Heart Association; LVEF, left ventricular ejection fraction; LVOT, left ventricular outflow tract; HCM, hypertrophic cardiomyopathy; LV, left ventricular.

**The corrected Table 1:**

**Table 1.** **Inclusion and exclusion criteria of EXPLORER-HCM and SEQUOIA-HCM clinical trials [4,5]**.

EXPLORER-HCM clinical trial investigating Mavacamten	
Key inclusion criteria	Key exclusion criteria
Aged at least 18 years	History of syncope or sustained ventricular tachyarrhythmia with exercise within the past 6 months
Diagnosed with obstructive HCM (LV hypertrophy with max LV wall thickness ≥15 mm or ≥13 mm if familial HCM)	QT interval corrected >500 ms using Fridericia’s formula
Peak LVOT gradient at least 50 mmHg (at rest, after Valsalva manoeuvre or exercise)	Paroxysmal or intermittent atrial fibrillation present on screening ECG
Left ventricular ejection fraction (LVEF) at least 55%	Persistent or permanent atrial fibrillation not on anticoagulation for 4 weeks+ or not adequately rate-controlled within the past 6 months
NYHA class II–III symptoms and able to perform upright CPET	Patients were allowed to continue standard hypertrophic cardiomyopathy medical therapy except disopyramide, including monotherapy with β blockers or calcium channel blockers, if dosing remained stable for at least 2 weeks before screening and no changes were anticipated during the study.
SEQUOIA-HCM clinical trial investigating Aficamten	
Key inclusion criteria	Key exclusion criteria
Adults between 18 and 85 years of age at screening	Moderate-severe valvular aortic stenosis and/or regurgitation. Moderate-severe mitral regurgitation not caused by systolic anterior motion of the mitral valve
BMI <35 kg/m^2^	Known or suspected infiltrative, genetic, or storage disorder causing cardiac hypertrophy that mimics HOCM (e.g., Noonan syndrome, Fabry disease, amyloidosis)
Has an end-diastolic LV wall thickness as measured by the echocardiography core laboratory of • ≥15 mm in ≥1 myocardial segment OR • ≥13 mm in ≥1 wall segment and a known-disease-causing gene mutation or positive family history of HCM	History of LV systolic dysfunction (LVEF <45%) or stress cardiomyopathy at any time during the clinical course of the disease
RER ≥1.05 and pVO2 ≤90% predicted on the screening CPET per the core laboratory	Inability to exercise on a treadmill or bicycle (e.g., orthopedic limitations)
Resting LVOT gradient ≥30 mmHg AND post-Valsalva LVOT-G ≥50 mmHg	Has been treated with septal reduction therapy (surgical myectomy or percutaneous alcohol septal ablation) or plans for either treatment during the study period
LVEF ≥60% at screening	History of syncope or sustained ventricular tachyarrhythmia with exercise within 6 months before screening
NYHA functional class II or III at screening.	Has received prior treatment with aficamten or mavacamten
Patients on beta-blockers, verapamil, diltiazem, or disopyramide should have been on stable doses for >6 weeks prior to randomization and anticipate remaining on the same medication regimen during the study. Patients treated with disopyramide must also be concomitantly treated with a beta-blocker and/or calcium-channel blocker	Paroxysmal atrial fibrillation documented during the screening period
Hemoglobin ≥10 g/dL at screening	Paroxysmal or permanent atrial fibrillation requiring rhythm restoring treatment (e.g., direct-current cardioversion, atrial fibrillation ablation procedure, or antiarrhythmic therapy) ≤6 mo before screening (This exclusion does not apply if atrial fibrillation has been treated with anticoagulation and adequately rate-controlled for >6 mo).

CPET, cardiopulmonary exercise stress testing; ECG, electrocardiogram; HOCM, hypertrophic obstructive cardiomyopathy; NYHA, New York Heart Association; LVEF, left ventricular ejection fraction; LVOT, left ventricular outflow tract; HCM, hypertrophic cardiomyopathy; LV, left ventricular.

This has been corrected as of June 4, 2026. The authors apologize for these errors.
